# The interaction between no folic acid supplementation during early pregnancy and preeclampsia increased the risk of preterm birth

**DOI:** 10.15537/smj.2023.44.3.20220695

**Published:** 2023-03

**Authors:** Yi-Jie Zhang, Hong Jiang, Chengqiu Lu, Yi Sun, Shudong Cui, Chao Chen

**Affiliations:** *From the Department of Neonatology (Zhang, Jiang), Affiliated Hospital of Qingdao University, Qingdao; from the Department of Neonatology (Chen), Children’s Hospital of Fudan University; from the Division of Neonatology (Lu), Gynecology and Obstetrics Hospital of Fudan University, Shanghai; from the Department of Neonatology (Sun), The Second Affiliated Hospital of Guangzhou Medical University, Guangzhou; from the Department of Neonatology (Cui), First Affiliated Hospital Nanjing Medical University, Nanjing, Chin.*

**Keywords:** preterm birth, folic acid supplementation, preeclampsia, interaction, risk factor

## Abstract

**Objectives:**

To explore if there is a positive additive interaction between no folic acid (FA) supplementation in early period of pregnancy and preeclampsia which increases the risk of preterm birth (PTB).

**Methods:**

We matched 1471 women who had live-birth singleton preterm infants with 1471 women who had live-birth singleton term infants at 15 Chinese hospitals in 2018. We excluded women who took folic acid less than 0.4 mg/d or less than 12 weeks in early stage, women with gestational hypertension, chronic hypertension, or preeclampsia during previous pregnancy. We calculate odds ratios for PTB by performing conditional logistic regression comparing preterm group with term group.

We quantified the interaction between 2 exposures by synergy (S) and relative excess risk due to interaction (RERI).

**Results:**

Approximately 40% of preterm cases did not take FA in early pregnancy. After adjusting confounding factors by logistic regression, when the 2 exposures (no early FA supplementation and preeclampsia) co-existed, the risk of all PTB increased significantly (aOR11=12.138; 95% CI 5.726-25.73), the interaction between 2 exposures was positive (S=1.27) and increased 2.385-fold risk of all PTB (RERI=2.385); and there were similar results on iatrogenic PTB (aOR11=23.412; 95% CI 8.882–60.71, S=1.18, RERI=3.347).

**Conclusion:**

Our multicenter study showed, for the first time, that there was a positive additive interaction between no FA supplementation in early pregnancy and preeclampsia which increased the risk of all PTB, especially iatrogenic PTB.

**P**reterm birth (PTB) is defined as birth before 37 weeks of gestational age and represents a major cause of death in newborns and children under 5 years of age.^1-3^ Based on clinical intervention, preterm births can be divided into iatrogenic preterm births and spontaneous PTBs.^1^

The etiology of PTB is thought to be multifactorial and is still largely unknown. Many studies have demonstrated that periconceptional folate acid (FA) supplementation could decrease the risk of PTB.^4-7^ Preeclampsia is also strongly associated with PTB.^1,8,9^ However, we do not know if the risk of preterm birth would increase even more if a pregnant woman lacked FA supplementation during the first 12 weeks of pregnancy and then went on to develop preeclampsia. There are very few studies relating to the interaction between no folic acid (FA) supplementation in early period of pregnancy and preeclampsia on PTB.

An interaction is defined as the combined effect caused by 2 or more exposure factors at the same time that is not equal to the sum of the individual effects. When the former is greater than the latter, it is defined as a positive interaction, thus indicating that the effect is enhanced when 2 or more factors co-exist at the same time; this means biological synergy. A synergy index (S) >1 indicates that there is a positive additive interaction between 2 exposure factors. Attributable interaction refers to the amount of effect caused by the interaction between 2 factors. Excess relative risk of interaction effects (RERI) is used to describe the relative effect size caused by attributive interaction. The larger the RERI is, the stronger the interaction between factors is.^10^

In the current study, we hypothesized that there is a positive additive interaction between a lack of FA supplementation during the early period of pregnancy and preeclampsia which increase the risk of PTB. The findings of this study provide new insights into our understanding of FA intake in early pregnancy and how this strategy might reduce the risk of PTB for women developing preeclampsia.

## Methods

A retrospective multicenter 1:1 matched case-control study was carried out. The data used in the current study were extracted from a ‘preterm risk factor study’ database.^11^ The ‘Preterm risk factor study was a case-control study carried out between January 2018 and December 2018 in 15 hospitals across China. The study included 15 hospitals: 4 county-level hospitals, 6 prefecture-level hospitals and 5 provincial hospitals from North-Western, Eastern, South-Central, and regions of China. Children’s Hospital of Fudan University served as the research center and was responsible for data coordination and the integration of information. The study was approved by the Research Ethics Committee of Children’s Hospital of Fudan University ([2018] no.: 84).

Selected cases were defined as mothers who gave birth to a live-born singleton preterm newborn (24-36 weeks). Controls were defined as mothers who delivered a live-born singleton term newborn (37-41 weeks and birth weight between 2500-4000 g). An eligible control was matched to a case by delivery date (within 2 days), delivery site (same hospital), and the newborn’s gender. The exclusion criteria were as follows: i) pregnant women who did not have a complete set of information relating to preeclampsia and the supplementation of folic acid; ii) pregnant women who took <0.4 mg/day of folic acid or <12 weeks during early pregnancy; iii) pregnant women who had chronic hypertension, gestational hypertension or preeclampsia during previous pregnancy, and iv) pregnant women who refused to participate.

The determination of gestational age was based on calculating the last menstrual period (irregular menstruation combined with ultrasonic examination). Maternal diseases were diagnosed by referring to the 9th Edition of “Obstetrics and Gynecology” (edited by Xie et al).^12^ Diabetes mellitus during pregnancy and preeclampsia were diagnosed according to national guidelines.^13,14^ Smoking included active smoking and passive smoking defined according to World Health Organization (WHO) definitions.^15^ Pre-pregnancy body mass index (BMI) was divided into 2 groups (<24 kg/m^2^ and ≥24 kg/m^2^) according to Chinese standards.^16^

We recorded a range of information relating to maternal and fetal characteristics, including maternal age, parity, maternal education, race, residence in pregnancy, family income, supplementation of folic acid (dose and duration), preeclampsia, supplementation of FA before pregnancy (dose and duration), smoking in pregnancy, previous preterm birth, diabetes mellitus in pregnancy, chronic renal disease, hypothyroidism, placenta problems, pre-pregnancy BMI, assisted reproduction, prenatal examination, and family history of hypertension.

This study is a 1:1 group case-control study. The parameters were set according to the odds ratio (OR) value of previous studies and the incidence of the control group, α=0.05, β=0.20, using pass 15.0 software to calculate the main risk factors. The sample size of preeclampsia (OR=9, 1.5% in the control group) was 26 cases. The sample size of FA supplementation (OR=1.3, 70% in the control group) was 1086 cases.

### Statistical analyses

We used Epidata version 3.4 (The EpiData Association, Odense, Denmark) to establish the database, perform logical consistency checks, and verify data. Stata version 15.0 (StataCorp, College Station, TX, USA) statistical software was used for statistical processing. Univariate analyses included the Chi-squared test, 2-sample independent t-tests, and the rank-sum test. A P-P plot was used to test normality. We used conditional multivariate logistic regression analysis to estimate the combined and individual adjusted odds ratios (aORs) of 2 exposures (no FA supplementation and preeclampsia) on the risk of PTB and stratified to estimate combined and individual adjusted odds ratios (aORs) of the 2 exposures on the risk of iatrogenic PTB and spontaneous PTB. Confounding factors were controlled for. *P*<0.05 was significant. The synergy index (S), relative excess risk due to interaction (RERI), and proportion attributable to interaction (AP) were calculated to estimate the interaction between 2 exposures.^10^ We used the combined and individual adjusted OR (aOR) values to calculate S, RERI, and AP.^10^ Missing data was deleted.

## Results

**Figure 1:**
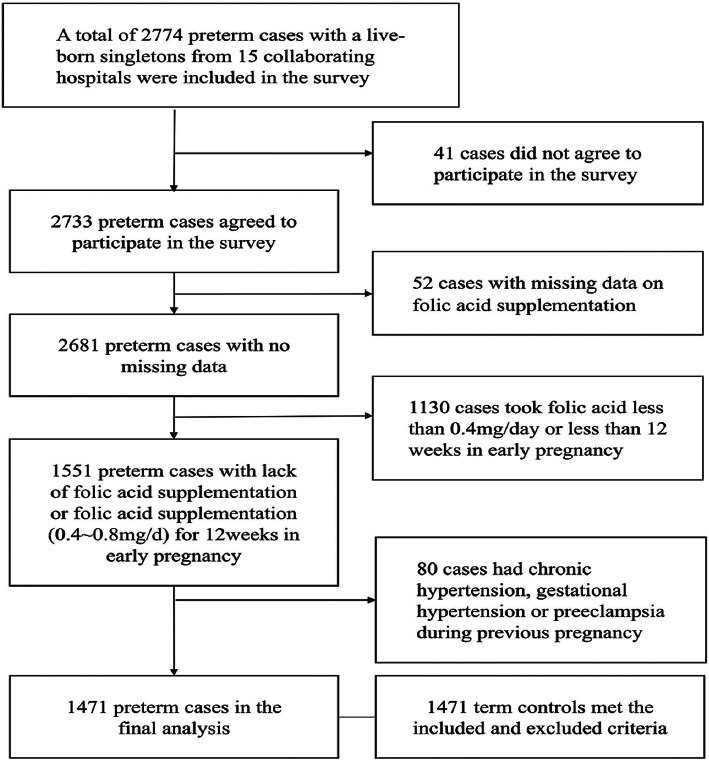
- Flowchart showing the recruitment process for participants.

Figure 1 shows a flowchart showing how the participants were recruited. A total of 2774 preterm cases were recruited initially. Of these, 41 cases did not agree to participate in the survey, 52 cases were missing data relating to folic acid supplementation, 1130 cases did not take 0.4 - 0.8 mg/day of folic acid for 12 weeks during early pregnancy, 80 cases had chronic hypertension, gestational hypertension or preeclampsia during previous pregnancy; therefore, these cases were all excluded. Consequently, there was a total of 1471 preterm cases in the case group; 58.3% (857 cases) delivered male infants. In total, 655 cases of preterm birth presented with iatrogenic preterm birth (iPTB) and 816 cases of preterm birth presented with spontaneous preterm birth (sPTB). Overall, the control group included 1471 term controls, who met the inclusion and exclusion criteria; finally, 58.3% (857 cases) delivered male infants.

Table 1 shows the demographic characteristics of the 2 groups. The mean age of all participants was 30.20±5.20 years. During pregnancy, 398 (13.5%) participants resided in the countryside, 1062 (36.1%) participants in small towns, and 1482 (50.4%) participants in cities. There were 787 (26.7%) participants with an education level of middle school or below, 688 (23.4%) of high school, and 1467 (49.9%) of university or above. The 2 groups showed significant differences (*p*<0.05) in terms of maternal age, parity, race, residence during pregnancy, maternal education, and family income monthly per person.

**Table 1:** - Demographic characteristics of the study participants.

Characteristics	Total n=2942	Preterm n=1471	Term n=1471	t/*P*
***Maternal age*** *	30.20±5.202	30.50±5.589	29.89±4.765	<0.001
<20	56 (1.9)	40 (2.7)	16 (1.1)	<0.001
20-34	2252 (76.5)	1075 (73.1)	1177 (80.0)	
≥35	634 (21.6)	356 (24.2)	278 (18.9)	
***Parity***			
Unipara	1448 (49.2)	658 (44.7)	790 (53.7)	<0.001
Multipara	1494 (50.8)	813 (55.3)	681 (46.3)	
***Race***			
Han	2777 (94.4)	1373 (93.3)	1404 (95.4)	0.013
Others	165 (5.6)	98 (6.7)	67 (4.6)	
***Maternal Education***			
Middle school or below	787 (26.7)	467 (31.7)	320 (21.8)	<0.001
High school	688 (23.4)	377 (5.6)	311 (21.1)	
University or above	1467 (49.9)	627 (42.6)	840 (57.1)	
***Residence during pregnancy***			
City	1482 (50.4)	650 (44.2)	832 (56.6)	<0.001
Small town	1062 (36.1)	568 (38.6)	494 (33.6)	
Countryside	398 (13.5)	253 (17.2)	145 (9.8)	
***Family income monthly per person (yuan)***			
<2,000	224 (7.6)	141 (9.6)	83 (5.6)	<0.001
2,000-10,000	2115 (71.9)	1053 (71.6)	1062 (72.2)	
>10,000	603 (20.5)	277 (18.8)	326 (22.2)	

Values are presented as numbers and percentages (%).

* mean±standard deviation

We carried out univariate analyses to compare the rate of prenatal diseases, FA supplementation, maternal age, and pre-pregnancy BMI between preterm and term groups (Table 2). In total, 1.5% of women in the term group had preeclampsia; this compared to 13.5% of women in the preterm group. In total, 31.0% of women in the term group did not take folic acid supplementation in the first trimester; this compared to 40.0% of women in the preterm group.

**Table 2:** - Results arising from univariate analysis of the risk factors for PTB.

Characteristics	Total n=2942	Preterm n=1471	Term n=1471	*P*-value
***Supplementation of FA in early pregnancy***			
No	1045 (35.5)	589 (40.0)	1456 (31.0)	<0.001
Full	1897 (64.5)	882 (60.0)	1015 (69.0)	
***Preeclampsia***			
No	2721 (92.5)	1272 (86.5)	21449 (98.5)	<0.001
Yes	221 (7.5)	199 (13.5)	122 (1.5)	
***Smoking in pregnancy***			
No	2758 (93.7)	1354 (92.0)	1404 (95.4)	<0.001
Yes	184 (6.3)	117 (8.0)	67 (4.6)	
***Previous preterm birth***			
No	2830 (96.2)	1377 (93.6)	1453 (98.8)	<0.001
Yes	112 (3.8)	94 (6.4)	18 (1.2)	
***Diabetes mellitus in pregnancy***			
No	2438 (82.9)	1176 (79.9)	1262 (85.8)	<0.001
Yes	504 (17.1)	295 (20.1)	209 (14.2)	
***Chronic renal disease***			
No	2940 (99.9)	1469 (99.9)	1471 (100)	0.157
Yes	2 (0.1)	2 (0.1)	0 (0)	
***Hypothyroidism***			
No	2790 (94.8)	1398 (95.0)	1392 (94.6)	0.617
Yes	152 (5.2)	73 (5.0)	79 (5.4)	
***Placenta problems***			
No	2569 (88.2)	1194 (81.2)	1402 (95.3)	<0.001
Yes	346 (11.8)	277 (18.8)	69 (4.7)	
***Pre-pregnancy BMI (kg/m^2^)***			
<24	2396 (81.4)	1178 (80.1)	1218 (82.8)	0.058
≥24	546 (18.6)	293 (19.9)	253 (17.2)	
***Assisted reproduction***			
No	2744 (93.3)	1363 (92.7)	1381 (93.9)	0.185
Yes	198 (6.7)	108 (7.3)	90 (6.1)	
***Prenatal examination***			
No	216 (7.3)	97 (6.6)	119 (8.1)	0.120
Yes	2726 (92.7)	1374 (93.4)	1352 (91.9)	
***Supplementation of folic acid before pregnancy***			
No	1592 (54.1)	859 (58.4)	733 (49.8)	<0.001
Partial	501 (17.0)	229 (15.6)	272 (18.5)	
Full	849 (28.9)	383 (26.0)	466 (31.7)	
***Family history of hypertension***			
No	2864 (97.3)	1427 (97.0)	1437 (97.9)	0.251
Yes	78 (2.7)	44 (3.0)	34 (2.3)	
Values are presented as numbers and percentages (%). PTB: preterm birth, FA: folic acid, BMI: body mass index				

The results arising from multivariate analysis are shown in Table 3; this included all statistically significant factors identified by univariate analyses, along with pre-pregnancy BMI and assisted reproduction, comparing all preterm births with term births. After adjusting for confounding factors preeclampsia (aOR=9.684, 95% confidence interval [CI]: [5.967-15.716]) were significantly associated with all preterm birth. Using full FA supplementation as a reference, the results demonstrated that no FA supplementation significantly increased the risk of all PTB (aOR=1.351, 95% CI: [1.073-1.701]), after adjusting for confounding factors.

**Table 3:** - Multivariate conditional logistic regression analysis of risk factors for all preterm birth (PTB).

Variable	aOR Value	95% CI	*P*-value
***Maternal age***		
<20	1.039	0.835-1.293	0.732
20-34	1.000		
≥35	2.136*	1.101-4.142	0.025
***Parity***		
Multipara	1.230*	1.039-1.507	0.030
***Race***		
Han	1.000		
Others	1.387	0.902-2.133	0.160
***Maternal education***		
Middle school or below	1.459*	1.122-1.898	0.005
High school	1.407*	1.126-1.759	0.003
University or above	1.000		
***Residence during pregnancy***		
City	1.000		
Small town	1.352*	1.094-1.670	0.005
countryside	1.686*	1.212-2.346	0.002
***Family income monthly per person (yuan)***			
<2,000	1.037	0.680-1.582	0.725
2,000-10,000	0.996	0.966-1.220	0.770
>10,000	1.000		
***Supplementation of FA in early pregnancy***			
No	1.351*	1.073-1.701	0.011
Full	1.000		
***Preeclampsia***		
Yes	9.684*	5.967-15.716	<0.001
***Smoking in pregnancy***		
Yes	1.691*	1.168-2.447	0.007
***Previous preterm birth***		
Yes	4.566*	2.620-7.959	<0.001
***diabetes mellitus in pregnancy***		
Yes	1.431*	1.146-1.786	0.002
***Placenta problems***		
Yes	11.634*	6.661-20.320	<0.001
***Pre-pregnancy BMI (kg/m^2^)***		
≥24	0.870	0.699-1.084	0.204
***Assisted reproduction***		
Yes	1.235	0.878-1.740	0.226
***Supplementation of folic acid before pregnancy***			
No	1.068	0.927-1.231	0.360
Partial	0.987	0.837-1.160	0.880
Full	1.000		

Conditional logistic regression analysis was performed. The control group was the reference group.

* *p*<0.05 was significant. aOR value and 95% CI after adjusting other confounders (including parity, maternal age, race, maternal education, residence during pregnancy, family income monthly per person, FA supplementation in early pregnancy, FA supplementation before pregnancy, preeclampsia, smoking in pregnancy, previous preterm history, diabetes mellitus in pregnancy, and placenta problems, pre-pregnancy BMI, and assisted reproduction). CI: confidence interval, BMI: body mass index, aOR: adjusted odds ratio, FA: folic acid

There was a total of 1471 preterm cases in the preterm group; 655 cases of preterm birth presented with iPTB and 816 cases of preterm birth presented with spontaneous preterm birth (sPTB).

Table 4 showed the results of individual and joint effects of no FA supplementation and preeclampsia on all PTB, subgroups (iPTB, sPTB), and measures of interaction.

**Table 4:** - Individual and joint effects of no FA supplementation and preeclampsia on PTB and measures of interaction.

Variable	Case/control number	aOR value	95% CI	*P*-value	RERI	AP	S
***All preterm birth (n=1471)/control group (n=1471)***						
None OR_00_	784/1001	1.000					
No FA supplementation OR_01_	488/448	1.290*	1.026-1.623	<0.001			
Preeclampsia OR_10_	98/14	9.463	5.090-17.60	0.027			
No FA supplementation + preeclampsia OR_11_	101/8	12.138*	5.726-25.73	<0.001	2.385	0.19	1.27
***Iatrogenic preterm birth (n=655)/paired control group (n=655)***							
None OR_00_	304/452	1.000					
No FA supplementation OR_01_	165/190	1.385	0.938-2.047	0.180			
Preeclampsia OR_10_	91/8	19.680*	8.462-45.77	<0.001			
No FA supplementation + preeclampsia OR_11_	95/5	23.412*	8.882-60.71	<0.001	3.347	0.14	1.18
***Spontaneous preterm birth (n=816)/paired control group (n=816)***							
None OR_00_	480/549	1.000					
No FA supplementationOR_01_	323/258	1.353*	1.009-1.814	0.043			
Preeclampsia OR_10_	7/6	1.140	0.317-4.106	0.841			
No FA supplementation + preeclampsia OR_11_	6/3	2.056	0.471-8.984	0.338	-	-	-

Conditional logistic regression analysis was carried out. The control group was the reference group. Taking preeclampsia and folic acid supplementation in early pregnancy as independent variables, patients were divided into 4 groups: no preeclampsia and folic acid supplementation (0.4-0.8 mg/d for 12 weeks) in early pregnancy (OR_00_), no preeclampsia and no FA supplementation in early pregnancy (OR_01_), preeclampsia and folic acid supplementation (0.4-0.8 mg/d for 12 weeks) in early pregnancy (OR_10_), preeclampsia and no FA supplementation in early pregnancy (OR_11_), no preeclampsia and FA supplementation (0.4-0.8 mg/d for 12 weeks) in early pregnancy (OR_00_) were used as the reference group. Adjusted ORs (aOR) were calculated by adjusted confounding factors, maternal age, parity, maternal education, residence, smoking in pregnancy, previous preterm birth, diabetes mellitus in pregnancy, placenta problems, supplementation of folic acid before pregnancy, assisted reproduction, pre-pregnancy BMI.

* *p*<0.05 was significant.FA: folic acid, BMI: body mass index, RERI: excess relative risk of interaction effect, AP: proportion attributable to interaction, S: synergy index, CI: confidence interval

After adjusting confounding factors conditional multivariate analysis revealed that no early FA supplementation (aOR_01_=1.290, 95% CI: [1.026-1.623]), preeclampsia (aOR10=9.463, 95% CI: [5.090-17.60]) was significantly associated with all preterm birth. When the 2 exposures (no early FA supplementation and preeclampsia) existed, the risk of all PTB increased significantly (aOR11=12.138; 95% CI: [5.726-25.73]). The interaction between 2 exposures was positive (S=1.27>1) and increased 2.385-fold risk of all PTB (RERI=2.385).

A total of 655 iatrogenic preterm births were compared with their paired control group. After adjusting confounding factors conditional multivariate analysis revealed that preeclampsia (aOR10=19.680, 95% CI: [8.462-45.77]) was significantly associated with iatrogenic preterm birth. When the 2 exposures (no early FA supplementation and preeclampsia) existed, the risk of iatrogenic PTB increased significantly (aOR11=23.412; 95% CI: [8.882-60.71]). The interaction between the 2 exposures was positive (S=1.18>1) and increased 3.347-fold risk of iatrogenic PTB (RERI=3.347).

A total of 816 spontaneous preterm births were compared with the paired control group. Following the adjustment of confounding factors, no early FA supplementation was significantly associated with spontaneous PTB (aOR_01_=1.353, 95% CI: [1.009-1.814]). However, preeclampsia was not significantly associated with spontaneous PTB (aOR11=1.140, 95% CI: [0.317-4.106], *p*=0.841). The combined effect of no early FA supplementation and preeclampsia was not significantly associated with spontaneous PTB (aOR11=2.056, 95% CI: [0.471-8.984], *p*=0.338).

## Discussion

The current study found that there was a positive additive interaction between no early FA supplementation and preeclampsia which increased the risk of all PTB (including iatrogenic PTB and spontaneous PTB) and iatrogenic PTB. The interaction between the 2 exposures increased 2-fold risk of all PTB (RERI=2.385) and 3-fold risk of iatrogenic PTB (RERI=3.347). Based on a review of the published literature, our study is the first to report the positive interactive effects between no early FA supplementation and preeclampsia which increased the risk of PTB. The possible mechanisms underlying this positive interaction could be partially explained by the fact that women who did not take FA may have high levels of Hct, a condition that is associated with hypertension.^17,18^ Our study may be representative of China because of the large sample size and the coverage of various development areas with different levels of economic development (East, middle, and West). Because this was a matched case-control study, we adjusted the confounding factors for preterm birth, such as the gender of the baby, season, environmental factors, maternal age, parity, residence, maternal education, smoking in pregnancy, previous preterm birth, diabetes mellitus in pregnancy, placenta problems, pre-pregnancy BMI, assisted reproduction, and supplementation of folic acid before pregnancy; consequently, our results are stable.

In agreement with other previous reports, our data confirmed that preeclampsia was significantly associated with PTB and iatrogenic PTB.^9,19^ The incidence of hypertensive pregnancy disorders reported in previous literature from China was between 2% to 12%.^20,21^ The incidence of preeclampsia in this study was 7.5% and was therefore relatively high.

We noted that a lack of FA supplementation in early period of pregnancy led to a significant increase in the risk of spontaneous PTB after adjusting for confounding factors. Similar to our findings, Shao et al^22^ also reported that the possibility of spontaneous PTB without premature rupture of membranes decreased by 17% (OR=0.83, 95% CI: [0.70-0.97]) in mothers who took folic acid supplements for more than 3 months before pregnancy and throughout pregnancy. This study showed that there were still 35.5% of mothers who did not take FA supplements in early period of pregnancy. Considering the increased risk of iatrogenic PTB for women who suffered preeclampsia, and the increased risk for spontaneous PTB, the policy for folic acid supplementation during early pregnancy should be strongly recommended in China.

### Study limitations

We are also aware that our current study has some limitations that need to be considered. This was a retrospective case-control study; consequently, it is difficult to infer causality. Therefore, a prospective cohort study is needed in the future. In addition, we did not test the folic acid levels in the plasma of the mothers included in this study. Although the confounding factors were controlled, residual confounding factors could not be completely excluded.

In conclusion, our study suggested that there was a positive additive interaction between a lack of folic acid supplementation in early pregnant period and preeclampsia which increased the risk of all preterm birth, especially the risk of iatrogenic PTB. A lack of FA supplementation in early pregnant period increased the risk of spontaneous PTB. This highlighted the importance of FA supplementation in early pregnancy with regard to reducing the risk of PTB in pregnant women who developed preeclampsia and spontaneous PTB.
